# An endemic rat species complex is evidence of moderate environmental changes in the terrestrial biodiversity centre of China through the late Quaternary

**DOI:** 10.1038/srep46127

**Published:** 2017-04-10

**Authors:** Deyan Ge, Liang Lu, Jilong Cheng, Lin Xia, Yongbin Chang, Zhixin Wen, Xue Lv, Yuanbao Du, Qiyong Liu, Qisen Yang

**Affiliations:** 1 Key Laboratory of Zoological Systematics and Evolution, Institute of Zoology, Chinese Academy of Sciences, Beijing 100101, China; 2 State Key Laboratory for Infectious Diseases Prevention and Control, National Institute for Communicable Disease Control and Prevention, Chinese Center for Disease Control and Prevention, Beijing, 102206, China; 3 University of Chinese Academy of Sciences, Beijing, 100041, China

## Abstract

The underlying mechanisms that allow the Hengduan Mountains (HDM), the terrestrial biodiversity centre of China, to harbour high levels of species diversity remain poorly understood. Here, we sought to explore the biogeographic history of the endemic rat, *Niviventer andersoni* species complex (NASC), and to understand the long-term persistence of high species diversity in this region. In contrast to previous studies that have proposed regional refuges in eastern or southern of the HDM and emphasized the influence of climatic oscillations on local vertebrates, we found that HDM as a whole acted as refuge for the NASC and that the historical range shifts of NASC mainly occurred in the marginal regions. Demographic analyses revealed slight recent population decline in Yunnan and south-eastern Tibet, whereas of the populations in Sichuan and of the entire NASC were stable. This pattern differs greatly from classic paradigms of temperate or alpine and holarctic species. Interestingly, the mean elevation, area and climate of potential habitats of clade a (*N. excelsior*), an alpine inhabitant, showed larger variations than did those of clade b (*N. andersoni*), a middle-high altitude inhabitant. These species represent the evolutionary history of montane small mammals in regions that were less affected by the Quaternary climatic changes.

The Hengduan Mountains (HDM, approximately 600 thousands km^2^) in China is a region located on the eastern and southern edges of the Qinghai Tibetan Plateau (QHTP) and is ranked as one of the world’s biodiversity hotspot. These mountains exhibit prominent altitudinal variations (1000–6000 m) and represent one of the most geologically and ecologically diverse areas in the world^1^. This region acts as both a cradle and museum of plant and animal diversity^1^,^2^. It holds more than one-half of the extant species of mammals, birds and freshwater fishes in China^3^,^4^,^5^,^6^. The remarkable biodiversity of the HDM is largely the result of low extinction rates over the past few million years^7^,^8^. Due to the differentiation of living organisms, the eastern and southern regions of the HDM are considered to have been isolated refuges during glacial or interglacial periods at different historical time points of the late Quaternary^9^,^10^,^11^. Understanding the underlying mechanisms that maintain the long-term persistence of high species diversity and endemism in this region is now a crucial issue in evolutionary biology.

The fauna of the HDM includes a large number of endemic large mammals, such as the giant panda (*Ailuropoda melanoleuca*), the Asian elephant (*Elephas maximus*) and the Sichuan snub-nosed monkey (*Rhinopithecus roxellana*), which were the most common members of the “*Ailuropoda-Stegodon* fauna”, widely distributed in southern China during the middle to late Quaternary^12^,^13^,^14^,^15^. The dramatic population contraction and range shift of these large mammals are now the focus of recent research and conservation activities in China and worldwide^16^,^17^,^18^,^19^,^20^. Despite the fierce controversy that has persisted for decades, climate changes and the massive growth in human populations are generally considered to be the major causes of the dramatic population contraction and range shift of large mammals in the past two millennia^16^,^21^,^22^. However, very little attention has been paid to the evolutionary dynamics of endemic small mammals, which play crucial roles in terrestrial ecosystems. Detailed studies of their phylogenetic structure and demographic history may shed light on the historical environmental conditions that have allowed the long-term persistence of high species diversity and endemism in the HDM.

Tectonic movements play important roles in changing the temporal and spatial patterns of species abundance and diversity^23^,^24^. Moreover, climate fluctuations during the Quaternary are considered to be the major events that promoted evolutionary succession, shaped genetic and geographic structure, and influenced the demographic dynamics of organisms in different regions of the world^25^,^26^,^27^. It has been reported that these climatic oscillations not only caused the massive extinction of mammalian megafauna but also promoted diversity loss in small mammals^28^,^29^. Several studies have emphasized that smaller-bodied species were more likely to exhibit range contraction and elevational increase during climatic changes^30^,^31^. However, a recent study indicated that small mammals can behaviourally escape climate change^32^. This point of view is likely supported by the HDM of China. For example, Chevrier’s field mouse (*Apodemus chevrieri*)^33^, the Sichuan field mouse (*A. latronum*) and Irene’s mountain vole (*Neodon irene*)^34^ from this region have shown relatively stable demographic dynamics through the climatic fluctuations of the Quaternary. However, it is unclear how these montane small mammal species maintained stable population size when their contemporaries and congeneric species in other regoins of the world were undergoing dramatic population dynamics, such as the rapid population expansion of two Japanese wood mouse species, *A. argenteus* and *A. speciosus*^35^.

The rat genus *Niviventer* is one of the dominant taxa in the local mammal communities in southern China. The *N. andersoni* species complex (NASC) in this genus includes two nominated species, *N. excelsior* and *N. andersoni*, both of which are highly adapted to middle to high elevations^36^,^37^. Their distribution is primarily confined to the HDM, with fragmented populations in nearby mountains, such as Qinling and Shennongjia^37^. Misidentifications within the genus *Niviventer* are common in the recent literature and museum collections^37^,^38^,^39^,^40^,^41^,^42^,^43^. However, the entire NASC can be easily distinguished from congeneric species based on their larger body size compared with those of other species within *Niviventer*. In addition, the mandibular teeth rows are more than 7 mm long, and the last one-third of the tail is white^5^,^37^. These characteristics make it feasible to distinguish this species complex from other congeneric species, not only using specimens from extant populations but also using materials from fossil sites. In addition, the NASC appears to be a well-supported monophyletic clade in molecular systematics^36^,^39^,^40^,^41^,^42^,^43^,^44^. It is unclear how the overall phylogenetic structure, distribution range and demographic dynamics of the NASC have responded to historical environmental changes.

To characterize the responses of animals to Quaternary climatic fluctuations^45^, a classic paradigm of glacial range contraction and interglacial range expansion of temperate species, called the “expansion–contraction” model^46^, has been proposed. However, alpine and arctic species show a higher tolerance for cold environments, which has allowed them to maintain a wider range of suitable habitats and sustain a larger population during the cooling of glacials, where as significant range contraction and population decline occurred during the interglacials. Furthermore, this series of events has also been predicted to occur in future periods of global warming^47^,^48^. This pattern has only been found in a few extant mammal species, such as the American pika (*Ochotona princeps*)^49^,^50^,^51^, the Holarctic northern red-backed vole (*Myodes rutilus*)^52^, and the Anatolian ground squirrel (*Spermophilus xanthoprymnus*)^53^.

Based on the body size, lifestyle and distribution characteristics of the NASC, we proposed the following hypotheses. Hypothesis 1: Several studies have indicated that biological or ecological features of small mammal are closely linked to changes in climate^30^,^31^. During climate warming, the habitats of the NASC shifted upward, and the area of suitable habitat probably contracted dramatically. However, during climate cooling, the habitats of these species will shifted downward, and the area of suitable habitat increased, likely leading to population expansion of these small mammals. Hypothesis 2: Small mammals are less likely than large mammals to respond to climate change^32^. The environmental change that drove the local extinction and population contraction of larger mammals in the HDM may have not significantly influenced the NASC, allowing the distribution range and demographic dynamics of this species complex to remain stable in different historical periods. However, species with higher elevation ranges are more likely to respond to climate changes^32^. The alpine inhabitant, *N. excelsior*, probably experienced more prominent range shifts and demographic fluctuations than its sister species, *N. andersoni* (a mid-high altitude inhabitant), during the climatic oscillations in the late Quaternary.

In the present study, we integrated fossil records with recent collection records (Fig. 1) of the NASC (Fig. 2A) and explored the phylogenetic structure and demographic history of this species complex based on three mitochondrial DNA fragments (Cytochrome b, CytB; Cytochrome oxidase subunit I, COI; the D-loop sequence, D-loop) and one nuclear fragment (the first exon of the interphotoreceptor retinoid binding protein gene, IRBP). Through these analyses, we sought to understand the evolutionary process of the NASC and reveal the underlying mechanisms that have allowed the long-term existence of high species diversity and endemism in the HDM.

## Results

### Fossil occurrences of the NASC

**Figure 1:**
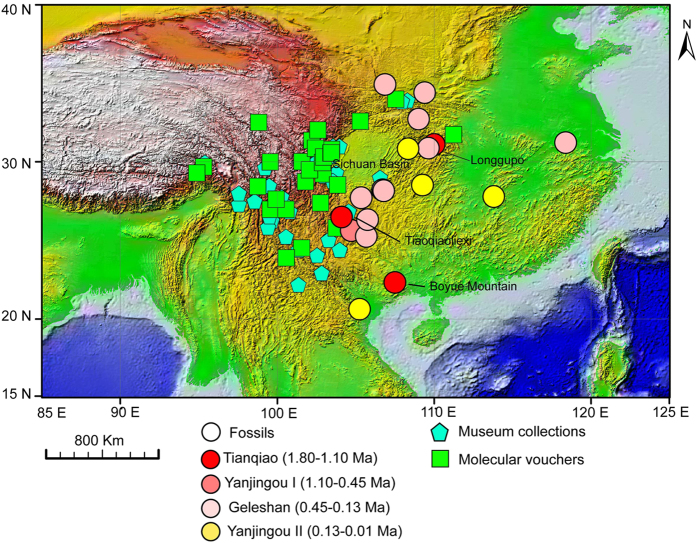
Distribution of the NASC as revealed by fossils, museum collections and molecular voucher specimens. Dots show fossil occurrences dating to different periods (represented by colour differences), blue pentagons show the locations of museum collections, and green squares show the locations of molecular voucher specimens. The presence data were mapped on the world topographic layer in ArcGis [9.0] (http://www.esri.com/software/arcgis/arcgis-for-desktop).

Compiling the fossil records of the NASC revealed 18 occurrences in southern China and one occurrence in Vietnam (Table S1). The earliest fossils of the NASC were unearthed from Lugupo, Wushan County, Chongqing, China, and are dated to the Tianqiao Period^54^. This location is much farther south than the distribution centre of the extant populations (Fig. 1). Two other early fossil records were unearthed from Tiaoqiaoliexi, Weining, Guizhou and Boyue Mountain, Chongzuo, Guangxi (Fig. 1 and Table S1). The largest number of fossil collections of the NASC were recorded from Pingba (Minimum number of individuals, MNI = 110) and Longgupo (MNI = 104) in Chongqing, China^54^ (Table S1). The available elevation records of these fossil sites ranged from 206 to 2000 m (Table S1). Most fossils of the NASC were from the Geleshan period (Fig. 1). These sites constitute the so-called “*Ailuropoda-Stegodon* fauna” and are well known for the occurrence of panda and stegodon, large mammals widely distributed in southern China during the Quaternary^55^,^56^. Four species of the genus *Niviventer* occurred in these sites: *N. fulvescens, N. confucianus, N. preconfucianus* and *N. andersoni*^54^. Notably, no fossils of the NASC have been found in the HDM. However, few fossil sites have been unearthed in this area. The fossils of the NASC in southern China were primarily unearthed from lower elevations; however, their current distribution is confined to higher mountains in south-western China (Fig. 1).

### Phylogenetic structure

**Figure 2:**
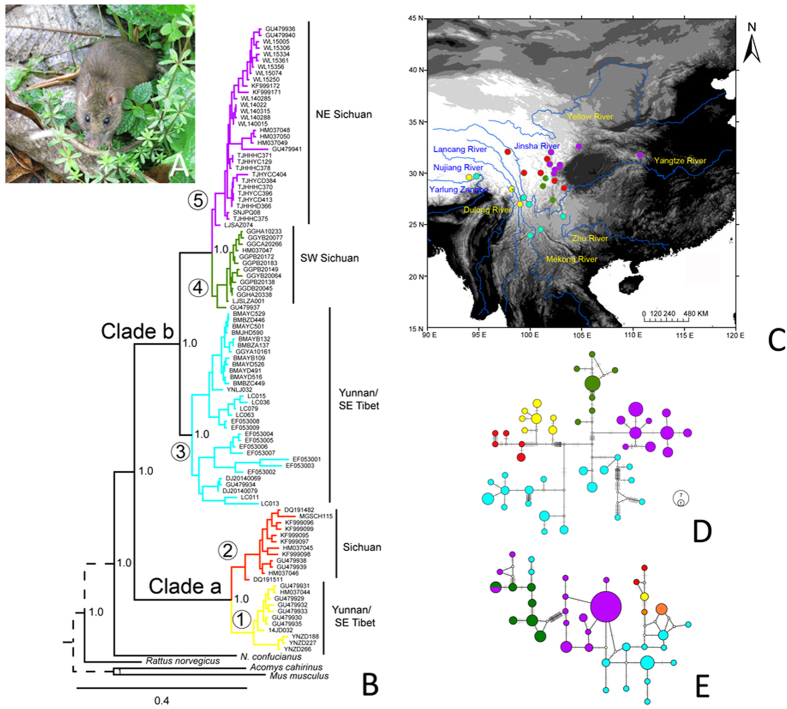
Phylogenetic structure of the NASC inferred from molecular data. (**A**) The profile of *N. andersoni* in the wild, photographed by D. Y. G., in Wolong, Sichuan (E 109.20, N 30.80). (**B**) Bayesian tree of the NASC inferred from the concatenated dataset of four DNA fragments. (**C**) Geographic distribution of five genetic lineages. The background of the map is classified by the values of the current altitude layer that is publicly available from Worldclim (http://www.worldclim.org/) and generated in ArcGIS [9.0] (http://www.esri.com/software/arcgis/arcgis-for-desktop). Major rivers within this region were drawn on the map in blue. Proportions of different genetic lineages at each sample site are given in different colors: yellow for lineage 1, red for lineage 2, blue for lineage 3, green for lineage 4 and purple for lineage 5. (**D**) Haplotype network inferred from CytB. (**E**) Haplotype network inferred from the phased sequences of IRBP. The size of cycles corresponds to the frequencies of different haplotypes, and the number of mutations is shown as links among different haplotypes. The colour of each genetic lineage (**C**,**D**,**E**) corresponds to the colours of the Bayesian tree (**B**). Small white circles represent haplotypes missing from the dataset.

In the Bayesian phylogenetic analyses of four DNA fragments, we identified two major clades (Fig. 2B). The overall structure was consistent with the current recognition of *N. excelsior* (=Clade a) and *N. andersoni* (=Clade b). *N. excelsior* included two lineages (1, 2): the first lineage was mainly from Yunnan and south-eastern Tibet, whereas the second lineage was from south-western Sichuan, mainly located at higher elevations. The phylogenetic structure within *N. andersoni* was similar to that of *N. excelsior*. The first lineage was from Yunnan and south-eastern Tibet (3). The second lineage was mainly from Sichuan. This Sichuan lineage was generally divided into the southern sub-lineage (4) and the northern sub-lineage (5) (Fig. 2C). This pattern indicated the parallel diversification of these two species in the HDM.

Ninety sequences of CytB revealed 46 haplotypes, which were defined by 217 polymorphic sites. The overall nucleotide diversity was 0.0354, with a haplotype diversity of 0.977. The genetic diversity of three mitochondrial DNA fragments is given in Table 1. The median-joining networks of CytB showed a prominent phylogeographic structure (Fig. 2D). Nuclear sequences contain ambiguous sites; therefore, we phased these sequences before conducting detailed analyses in DnaSP v5^57^. Thirty-eight haplotypes were identified from IRBP (Fig. 2E), with very good phylogenetic structure, albeit with some inconsistency with the mtDNA clades.

### Divergence time among genetic lineages

**Figure 3:**
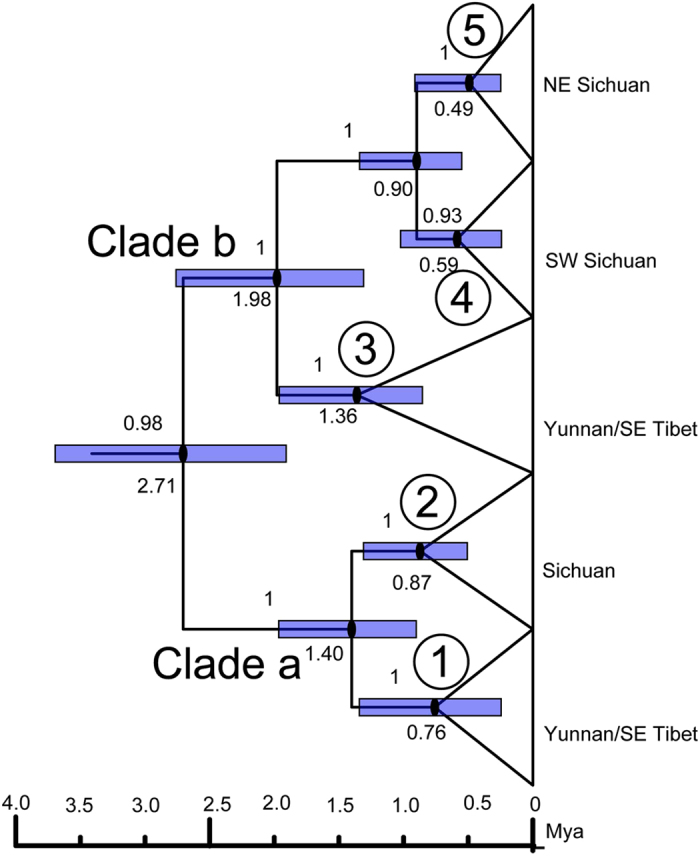
Divergence time of the NASC.Blue bars show the 95% confidence interval of divergence time at each node. Clades a and b correspond to *N. excelsior* and *N. andersoni* respectively. Arabic numbers correspond to the same genetic lineages in Fig. 1.

Molecular dating based on the concatenated dataset of the four DNA fragments (Fig. 3) revealed that the deepest divergence time within the NASC dated to approximately 2.71 Mya (3.70–1.90 as 95% HPD). The divergence time of lineages 1 and 2 within *N. excelsior* was dated to approximately 1.40 Mya (0.91–2.00 as 95% HPD). The divergence time of lineages 3–5 within *N. andersoni* was dated to approximately 1.98 Mya (2.77–1.30 as 95% HPD). The divergence time of lineages 4 and 5 was dated to approximately 0.90 Mya (1.36–0.54 as 95% HPD).

### Range shifts and demographic history

**Figure 4:**
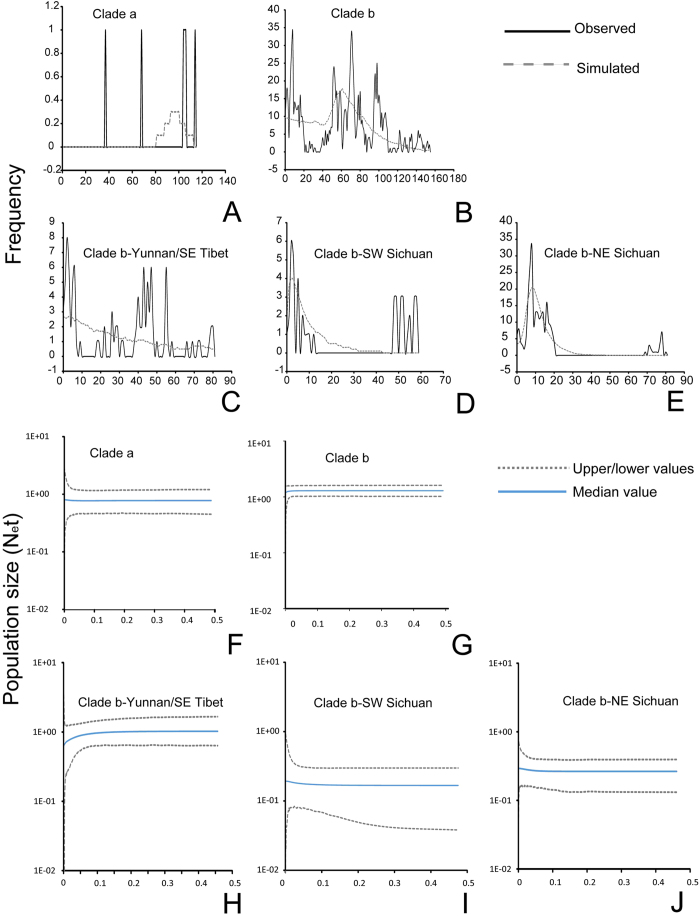
Demographic dynamics of the NASC inferred from mismatch distribution and the extended Bayesian skyline plots. (**A–E**) Mismatch distribution. (**F–J**) Extended Bayesian skyline plots. The demographic dynamics of *N. andersoni* are classified by different genetic lineages (**C**,**D**,**E**,**H**,**I**,**J**), corresponding to lineages 3, 4 and 5 in the Bayesian tree (Fig. 2B).

For both clades (a and b) and all five lineages (1–5) recovered in the Bayesian analyses, Fu’s Fs test resulted in low values with insignificant support, and Tajima’s D showed non-significant negative values for all mitochondrial regions (Table 1). The mismatch distributions for lineages showed a multimodal pattern, suggestive of demographic equilibrium and stable populations (Fig. 4A–E) with non-significant values for both the sum of the square deviation and raggedness index (Table 1). The EBSP results also demonstrated relatively stable population dynamics in clades a and b. A slight trend of recent population decline was identified in the lineage of Yunnan/SE Tibet in *N. andersoni* (Fig. 4F).

### Morphological variation of voucher specimens and taxonomic implication

According to collection records of 38 adult voucher specimens, the values for body weight (BW), head and body length (HBL), tail length (TL), hind foot length (HFL) and ear length (EL) of *N. andersoni* were similar to the original description for the holotype of *N. andersoni* (BMNH 11.2.1.135); the HBL (178 mm) for the original description of *N. excelsior* is considerably larger than that of molecular voucher specimens, which had values from 121 to 145 mm (Table 2). However, according to the original collection records of the holotype (BMNH, 11.2.1.131), the handwriting “148” likely was misidentified as “178” by Thomas^58^. Museum specimens with general measurements and skull morphology that fell well outside those of molecular vouchers and holotypes were excluded in the subsequent ecological niche modelling.

### Ecological niche modelling

**Figure 5:**
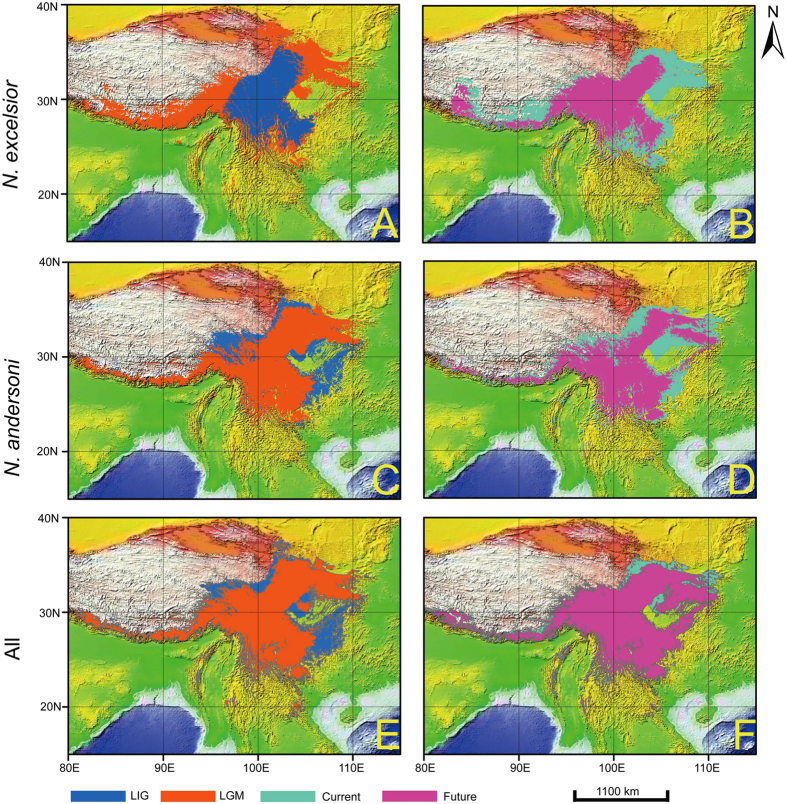
Ecological niche modelling of the NASC using 19 bioclimatic variables. The predicted potential distribution reclassified by MTPLT are given in different colors. The predicted potential distribution in different historical periods are mapped on the world topographic layer in ArcGis [9.0] (http://www.esri.com/software/arcgis/arcgis-for-desktop).

Under the modelled climatic conditions of the Last Interglacial (LIG), the suitable habitat of *N. excelsior* prominently contracted (Fig. 5A). A dominant trend of westward and northward expansion since the Last Glacial Maximum (LGM) (Fig. 5A). The pattern of the mid-Holocene was extremely similar to the present condition and hence is not shown in Fig. 5. The range of *N. excelsior* is predicted to contract substantially in the future (Fig. 5B). In contrast to *N. excelsior, N. andersoni* showed less range shifts in different historical periods (Fig. 5C,D). The ENM results revealed that the historical range shift of the whole NASC primarily occurred in the north-eastern, north-western and south-eastern HDM (Fig. 5E,F). The average test of AUC for the replicate runs was greater than 0.97 for all five periods, confirming the excellent predictive power of the models. The binomial probabilities (<0.001) for eleven common thresholds indicated that our predictions were significantly better than those of a random model.

The average elevation of *N. excelsior* was modelled to be the highest in the future (3424.25 ± 1200.84 m) and lowest during the LGM (2927.75 ± 1499.22 m) (Table 3). Additionally, the area of the potential habitats of this species was smallest in area during the LIG (about 506, 400 km^2^). During future warming (8.96 ± 6.25 C), *N. excelsior* is predicted to suffer prominent range contraction again (approximately 669, 100 km^2^) (Table 3). This pattern is comparable to that of typical alpine and Holarctic species. However, the central and southern regions of the HDM were identified as highly suitable habitats throughout all of the different historical periods. The average elevation of *N. andersoni* was highest during the future climate warming (2894.67 ± 1257.17 m) and lowest during the coldest LGM (2679.52 ± 1318.84 m), and its potential habitat was smallest during future warming (777, 200 km^2^) (Table 3). Interestingly, the potential range of the whole NASC showed little variation in either area or elevation through the late Quaternary (Table 3).

## Discussion

### Phylogeographic structure and divergence time of the NASC

In the present study, we found high levels of genetic diversity and clear patterns of phylogeographic structure in the NASC (Fig. 2B). Early diverged lineages of both of these two species were from Yunnan and Tibet (Fig. 2B). The earliest branches of both clades showed longitudinal dispersal in the contact regions of the QHTP and the HDM (Fig. 2C), particularly at the boundary between Yunnan and Tibet (lineage 1 in *N. excelsior* and lineage 3 in *N. andersoni*). The lineages from Yunnan/southeast Tibet (SE Tibet) and Sichuan were primarily separated by the Yangtze River. Within *N. andersoni*, lineage 4 from Southwest Sichuan (SW Sichuan) was confined to the Qionglai and Daxiangling Mountains, whereas the NE Sichuan lineage 5 extended from Minshan to Qinling. Interestingly, both *N. excelsior* and *N. andersoni* showed trends of northward expansion in geographical evolution. According to the distribution of these genetic lineages, we concluded that the mountain system probably shaped the geographic pattern of this species complex. This pattern is commonly recognized in alpine small mammals, such as pikas^49^ and chipmunks^59^. The upstream area of the Yangtze River (Jinsha River) contributed to the geographic structure of both clades. However, the isolation effect of this river is weakened by seasonal climate changes in which its upper stream is covered by ice in winter, as demonstrated by the presence of lineages 2 and 3 at both sides of the Jinsha River (Fig. 2B).

Previous studies revealed the divergence time of the *Niviventer* started in the late Miocene^36^,^44^. According to the molecular dating in the present study, the NASC probably originated during the transition of the late Pliocene to early Pleistocene around 2.71 Mya, approximately the same time that the average amplitude of global climate oscillations increased to 41-ka climate cycles^60^,^61^. For mid-high altitude species, climate warming could promote genetic isolation between different mountain systems, followed by specialization in local habitats. Subsequent climate cooling likely drove the NASC to climb down these mountains and allowed them to disperse and hybridize at lower altitudes. The divergence times of lineages 1 and 2 and lineages 3, 4, and 5 were dated back to 1.40 and 1.98 Mya, respectively. This divergence is probably attributable to geographic diversification after expansion. During this period, the upstream portion of the Yangtze River became the major geographic barrier between the lineages of Yunnan/SE Tibet and Sichuan. The divergence time of SE Sichuan and NE Sichuan was dated to approximately 0.90 Mya, during the Mid-Pleistocene transition, when the low-amplitude 41-ka climate cycles of the earlier Pleistocene were replaced by the high-amplitude 100-ka cycles of the Middle and Late Pleistocene^61^. This transition, known as the ‘mid-Pleistocene revolution^62^,^63^, most likely promoted environmental heterogenization in SE and NE Sichuan, and then induced genetic differentiation between lineage 4 and 5.

### Range shifts of the NASC

The NASC probably thrived from the early Pleistocene, when southern China was dominated by a wet climate, and the “*Ailuropoda-Stegodon* fauna” was well developed in heavily forested regions^64^. During this period, the sub-tropical mixed deciduous broad-leaved and coniferous forest, the preferred vegetation of the NASC, was widely distributed in southern China^64^,^65^. The multiple instances of northward expansion of temperate species during warm periods alternated with the southward expansion of alpine species during cold periods^55^. During the late Pleistocene, large mammals of the “*Ailiropoda-Stegodon* fauna” showed a prominent population decline, as documented by fossils and genomic data^66^,^67^. This population decline occurred at approximately the same time as the two largest Pleistocene glaciations in China, the Naynayxungla Glaciation (0.78–0.50 Mya) and the Penultimate Glaciation (0.30–0.13 Mya)^68^,^69^. Most fossil records of the NASC have been dated to the Penultimate Glaciation, during the Geleshan Period (0.45–0.13 Mya)^54^, which implies a slight range expansion of this species complex from the HDM to nearby mountainous regions, such as Guizhou and Chongqing (Fig. 1). However, the accuracy of assigning specimens from northern Vietnam, Guangxi and Anhui in China to the NASC (Table S1)^70^,^71^,^72^ requires further investigations.

According to the ENM results, the potential habitat of *N. excelsior* contracted into the central and south-eastern areas of the HDM during the last interglacial, approximately 0.13–0.075 Mya, (Fig. 5A). This contraction can be attributed to a warm climate (Table 3), which likely forced this species to shift to higher habitats. Pollen-based quantitative precipitation reconstruction in northern China revealed a gradual intensification of monsoons from 0.14–0.07 Mya. This climate change induced a dramatic decrease in precipitation and an abrupt change in vegetation in northern China^73^,^74^. This type of environment is unfavourable for herbaceous plants, which were important food resources for alpine small mammals, and most likely resulted in a range contraction of *N. excelsior*. By contrast, the range of *N. excelsior* expanded with a clear trend of westward extension to the edge of the southwestern QHTP- Himalaya region during the LGM (~0.025 Mya). This expansion may be attributable to a cooler climate that drove *N. excelsior* to low altitude and allowed it to expand to nearby regions. In contrast to *N. excelsior*, the range of *N. andersoni* was larger in the LIG than in the LGM, mainly in the northern Hengduan Mountain and the regions around the southern Sichuan Basin in Guizhou and Chongqing. The potential range of *N. andersoni* slightly contracted in the LGM with westward expansion in the Himalaya mountains. However, future climate warming will reduce the potential ranges of both species.

Compared with the northern and western HDM, large areas of the central and southern regions of the HDM likely were less affected by the Quaternary climatic fluctuations, and remained as highly suitable habitats for both species. These regions were characterized by the establishment of warmer, wetter and perhaps more seasonal conditions during the transition from the Pleistocene to Holocene^75^, during which a high level of plant diversity was preserved^1^,^76^. These high mountains and deep valleys weakened the influence of extreme climate changes and also bridged range shifts of small mammals during climatic oscillations. The overall range of the NASC showed relatively less variation compared with other montane small mammals in Europe and North America.

### Demographic dynamics of the NASC

Overall, climatic changes through the Quaternary did not significantly affect the population size of the NASC. These climate changes also had much less impact on the population size of *N. andersoni* than on *N. excelsior*. This finding is in agreement with hypothesis 2: the middle-high altitudes species complex NASC was not significantly affected by the Quaternary environmental changes. Species within the genera *Niviventer* not only live on the forest floor but also are highly adapted climbers and thus have a wide range of available resources in the forest. According to the resource-use hypothesis, during episodes of climate triggered habitat change, specialists are more prone to suffer from resource limitation^77^. Consequently, specialists are more susceptible to habitat fragmentation, vicariance, directional selection and extinction. Since environmental changes have stronger effects on biome specialists than on generalist species, which can find resources in different biomes, specialists are predicted to have higher speciation and extinction rates than generalist^78^. Occurrence data for the NASC showed that *N. andersoni* has a wider elevational range than *N. excelsior* (Table S2). During the recurrent environmental extremes, *N. excelsior* likely had higher rates of resource limitation and population decline than did *N. andersoni*.

Generally speaking, the value of MAT for the potential habitats of the whole NASC at the LGM was only 2–4 °C lower than the present conditions (Table 3), considerably weaker than the average deviation (5–9.6 °C) at the global scale^79^,^80^. This change corresponded to an elevation shift of less than 1000 m, which would be readily accommodated by a mid-high altitude montane small mammals, such as *N. andersoni* in the central and southern HDM, but less readily by high-elevation species, such as *N. excelsior*. The mean annual temperature in the potential habitats of the NASC in the LGM was approximately 1.55 °C lower than in the LIG and approximately 4.28 °C lower than under present conditions (Table 3). The mean annual precipitation in the potential habitats of the NASC in the LGM and LIG were slightly lower than under present conditions (Table 3), but these changes appear to have mainly affected the marginal region of the HDM. The central and southern regions were less affected by these environmental changes. The stability of historical environmental conditions in the HDM is an important contributor to the stable demographic history of mid-high altitude small mammals such as *N. andersoni*. These results also shed light on the high diversity of pikas (Lagomorpha: *Ochotona*), another group of alpine specialists surviving in HDM and nearby regions, whereas congeneric species in other regions of the world suffered widespread extintion or dramatic population contraction^49^,^50^,^51^. These stable historical enviromental conditions are also likely one of the major reasons for the long-term persistence of high species diversity and endemism in this region.

The recent population decline of the NASC in Yunnan and south-eastern Tibet revealed by the EBSP results highlights a trend towards environmental change in this region, including deforestation and habitat fragmentation caused by changes in land usages. Moreover, human populations in southern China have increased rapidly in the past two millennia^81^ and have likely influenced the range and population sizes of local montane mammals, such as the NASC. Moreover, the ENM results for future time periods demonstrated a clear trend of range contraction and habitat fragmentation in the central HDM. Prominent warming (more than 3 °C higher than present conditions) is predicted in the habitats of the NASC. Differentiation of the eastern and western regions of the HDM will become more prominent. Multiple fragmented populations in the marginal regions of the HDM probably will suffer population decline. These changes will present further challenges for biodiversity conservation in this region.

## Conclusions

The demographic history of the NASC is relatively stable, which may be attributable to moderate environmental changes in the central and southern regions of the HDM through the late Quaternary. The stable demographic history and the high suitability of the central and southern HDM can be partially attributed to moderate environmental fluctuations, as indicated by the relatively low amplitude of changes in temperature and precipitation in the HDM. Moreover, although the NASC is subjected to elevational limitations, these omnivores have relatively wider forage selection in forests, further allowing them to survive during extreme climatic transitions. We posit that these conditions, together with the striking geological heterogeneity of the HDM, are the chief factors underpinning the long-term persistence of high species biodiversity and endemism in this region. The influence of Holocene climatic oscillations on the demographic history of local fauna in the HDM likely was more prominent in alpine species compared with mid-high altitude species. However, the acceleration of climate changes in the future will likely affect the evolution dynamics of both species.

## Materials and Methods

### Occurrence data and molecular sampling

To explore the historical range of changes in the NASC, we categorized their occurrence into three groups: fossil records, museum collections and recent collections for molecular studies. All experimental protocols were approved by the Animal Care and Use Committee of the Institute of Zoology, Chinese Academy of Sciences.

For fossil records, we compiled the fossil locations of the NASC (Table S1 and supplementary references). The information was obtained from the Paleobiology Database (http://paleodb.org/cgi-bin/bridge.pl) and the National Infrastructure of Mineral Rock and Fossil Resources for Science and Technology of China (http://www.nimrf.net.cn/). We also searched zoological records from 1864 to 2015 (http://apps.webofknowledge.com/) and conducted an extensive search of the original literature. The Quaternary rodents of Sichuan and Guizhou supports the recognition of five periods: Taimiao (2.6–1.8 Mya), Tianqiao (1.8–1.1 Mya), Yanjingou I (1.1–0.45 Mya), Geleshan (0.45–0.13 Mya), and Yanjingou II (0.13–0.01 Mya)^54^. The relative abundance of fossils from each sites were quatified by the minimum number of individuals (MNI)^82^, a method commonly used in vertebrate palaeontology.

The information from museum collections of recent populations included data from the Natural History Museum in London (NHM); the Institute of Zoology, Chinese Academy of Sciences (IOZCAS); and the Kunming Institute of Zoology, Chinese Academy of Sciences (KMIOZCAS; partially available at http://www.kiz.cas.cn/zyfw/kxsjk/xndq/). Additionally, the information collected from specimens studied by Musser^83^ and provided by the Global Biodiversity Information Facility (http://www.gbif.org/) was included.

Recent occurrences of the genus *Niviventer* in China were verified by extensive sampling of murids, recently carried out by IOZCAS, and the National Institute for Communicable Disease Control and Prevention, Chinese Center for Disease Control and Prevention (ICDC). In total, approximately 2500 individuals of the genus *Niviventer* were collected. The proportion of the NASC counted by number of individuals within the mammal communities was quite low. After the exclusion of mistaken identifications of data from GenBank, a total of 99 isolates from 28 locations were identified as the NASC (Fig. 1, Table S2).

### DNA sequencing

Total genomic DNA was isolated from muscle or liver tissues using the Easy Pure Genomic DNA Kit (Transgene Biotech, Beijing, China). Three mitochondrial markers (CytB, COI, and D-loop) and one nuclear marker (IRBP) were amplified. The primers and their primary references are the same as those used in previous studies^36^,^84^,^85^,^86^,^87^. The amplifications were carried out in 25-μL reactions containing approximately 25 ng of extracted DNA (approximately 1 μL), 12.5 μL polymerase chain reaction (PCR) mix (containing 200 μM of each dNTP, 0.2 μM of each primer, and 0.75 unit of LA *Taq* polymerase), and 10.5 μL dd H_2_O. The sequencing primers were the same as those used in the amplifications. All PCR products were directly sequenced in both directions with an ABi 3100 automatic sequencer (Applied Biosystems, Foster City, California, USA) using the ABi PRISM BigDye Terminator Cycle Sequencing Ready Reaction Kit with AmpliTaq DNA polymerase (Applied Biosystems, Foster City, California, USA).

### Phylogenetic analysis and genetic diversity

We appended the new data generated in the present study to those used in our previous study and data from GenBank^36^,^40^,^41^,^42^,^43^,^88^ and reconstructed a phylogenetic tree using CytB and the concatenated sequences of the four DNA fragments. The sequences were aligned with ClustalW^89^ as implemented in MEGA^90^. We determined the substitution models of each codon position of CytB, COI and IRBP, and the whole sequence of the D-loop using Modelgenerator V 851^91^. Bayesian tree searches were performed under partitioned models in the parallel version of MrBayes 3.2.2^92^. We performed four independent runs of these datasets, with two cold and two heated Markov chains of five million generations. The trees were sampled every 1000 steps, and the first 25% of the trees was discarded as burn-in. Convergence was monitored using the standard deviation of split frequencies, and the sufficiency of sampling was checked using Tracer v 1.5^93^.

We analysed the genetic diversity of the NASC using three fragments of mitochondrial DNA. We calculated the nucleotide diversity (pi), the number of haplotypes (h) and the haplotype diversity (hd) based on the major phylogenetic lineages recovered in the above Bayesian analyses. These analyses were conducted in DnaSP v5^57^. Moreover, to infer the intraspecific phylogeny among haplotypes, the median-joining network of haplotypes for CytB and the phased IRBP was generated using the median-joining algorithm in NETWORK 4.6.1.1^94^. Nuclear sequences contain a large number of ambiguous sites; we phased these sites before submitting them to reconstruct the network relationship among haplotypes. We used the maximum parsimony option to remove excess links and median vectors^95^.

### Demographic analysis

We used multiple approaches to estimate the demographic dynamics of the NASC. First, we calculated Tajima’s *D*^96^ and Fu’s F statistics^97^ (with 10,000 permutations) to test the neutral equilibrium model of the evolution of major lineages. Significant and large negative values of Tajima’s D and F are indicative of population expansion, whereas positive values for these tests indicate a decrease in population size. For each test, we assessed significance by generating null distributions from 10,000 coalescent simulations of a neutrally evolving equilibrium model. CytB, COI, and D-loop were used in these analyses separately. Second, we tested the pairwise mismatch distribution for the signature of the demographic expansion using the concatenated dataset of the four DNA fragments. A unimodal mismatch distribution is apparent when the population experiences a sudden expansion. In contrast, multimodal and ragged mismatch distributions are indicative of a stable or contracting population. These analyses were conducted in Arlequin 3.1^98^.

Moreover, we explored the demographic history of major genetic lineages using extended Bayesian skyline plots (EBSPs), which were implemented in Beast 1.8.2^99^. The concatenated dataset of four DNA fragments was used. For the divergence rate of mitochondrial DNA and nuclear DNA in small mammals, most studies revealed values ranging from 5% to 1%^51^,^100^. Our previous study showed that the time of the split of the genus *Niviventer* from other rat genera was approximately 7.44 Mya^36^. The substitution rate of the genus *Niviventer* following Pesole *et al*.^101^ was estimated using V = K/2T (K is the sequence divergence for each pair of species group, and T is the divergence time), indicating rates of 2.40%, 1.04%, 1.96% and 0.56% (per Mya) for CytB, COI, D-loop and IRBP, respectively. A strict molecular clock model was used with substitution models, clock models and trees unlinked for mitochondrial and nuclear DNA fragments. For all lineages, analyses were executed for 10 million generations, sampling every 1000 steps and discarding the first 25% of the results as burn-in. We performed each analysis twice to test the convergence of the results. For all analyses, the effective sample size of all parameters exceeded 200.

### Dating the divergence time of genetic lineages

The divergence time among major lineages within the species complex *N. andersoni* was determined using Bayesian MCMC dating. We appended new data generated in the present study with those of a previous study^36^ and dated their interspecific and intraspecific divergence times using fossil calibration points. Two fossil calibrations were used: (1) the split of basal Murinae (12 Mya)^102^,^103^, as this date is frequently used as a fossil calibration point in dating the divergence of mammals, and (2) the divergence of *Apodemus* (10.4 Mya)^104^. The earliest fossils of the NASC were dated at approximately 1.8 Mya^54^. Nevertheless, there is distinct sample bias of fossil sites in south-western China; specifically, Sichuan, Yunnan and Tibet remain poorly explored; therefore, we did not use this date in calibration. This analysis was conducted in a parallel version of Beast 1.8.2^105^. The Yule speciation model was used. The first 25% of the trees in each run was discarded as the burn-in phase. Trees were summarized in TreeAnnotator 2.1.2^106^, and the effective sample sizes of parameters were checked with Tracer v1.5^93^.

### Morphological analysis

Due to the lack of comprehensive systematic studies, a large number of museum collections of the NASC in China were misidentified, which prevented the use of all of these data in ecological niche modelling. We calculated the mean values based on general measurements of voucher specimens included in the present study. These measurements included values of body weight (BW), head and body length (HBL), tail length (TL), hind foot length (HFL) and ear length (EL). Infants and sub-adults were excluded in the present study. By using these values and skin and skull morphology, we identified and filtered the museum collection records of the NASC. Only these records confirmed in the present study were included in the subsequent ecological niche modelling.

### Ecological niche modelling and analyses

The distribution of the confirmed museum collection records and molecular voucher specimens (Fig. 1) was used to predict the potential range of the NASC in different periods using maximum entropy modelling^107^. The geographic coordinates for each locality were obtained from the original databases, from the original records in the literature, or from Google Earth (http://www.google.com/earth/index.html). To visualize the effects of global climate change on the change in the range of the NASC, we reconstructed its historical (including LIG, LGM and mid-Holocene), current and future distributions. The original climate data of four periods (LGM, middle Holocene, current and future) were downloaded from the WorldClim database (http://worldclim.org) at 2.5-min resolution. The climate data of LIG were resampled to the same cell size as that of the other four datasets. These data included 19 standard bioclimatic parameters commonly used in ecological niche modelling. The occurrences of *N. excelsior, N. andersoni* and both species were used separately in modelling.

Primary analyses that included all 19 parameters and eight parameters (Bio 2, 4, 10, 11, 15, 17, 18, 19 by excluding the parameters that showed high autocorrelations) resulted in very similar patterns. Here, we used 19 parameters to ensure that our methods were comparable with those of most previous studies. We extracted these bioclimatic data in the regions of interest by defining a rectangle (N 0–45°, E 80–125°). Seventy-five percent of the occurrence data was randomly selected to construct models, and the remaining twenty-five percent was used to test the model. The number of maximum interactions was set to 2000. The probability of suitable environmental conditions for the species was quantified by values from 0 to 1 in each grid cell. The performance of the models was verified by averaging areas under the receiver operating characteristic curves (AUC) and the binomial probabilities over 10 replicate runs.

To investigate the elevation and range shifts of the NASC, we selected the minimum training presence logistic threshold (MTPLT) to reclassify the values of raster files: values lower than the MTPLT were classified as “0”, indicating an absence area, whereas values higher than the MTPLT were classified as “1”, suggesting a presence area^108^. The presence of *N. excelsior* and *N. andersoni* in the LIG and current time were mapped on the LGM and future separately to illustrate the range shifts that occurred in the different periods. The areas of presence were calculated separately for each of the five historical periods. Moreover, we assumed that the elevation of the HDM did not change through the late Quaternary and then extracted the elevations of the presence area in each of the five periods. We also extracted the values of mean annual temperature (MAT, Bio1) and precipitation (MAP, Bio12) within the presence area to quantify climatic changes in different periods. These analyses were performed using the ArcGIS [9.0] (ESRI, Redlands, CA) platform, installed at the Chinese Ecosystem Research Network, Institute of Botany, Chinese Academy of Sciences.

## Additional Information

**How to cite this article**: Ge, D. *et al*. An endemic rat species complex is evidence of moderate environmental changes in the terrestrial biodiversity centre of China through the late Quaternary. *Sci. Rep.* **7**, 46127; doi: 10.1038/srep46127 (2017).

**Publisher's note:** Springer Nature remains neutral with regard to jurisdictional claims in published maps and institutional affiliations.

## Supplementary Material

Supplementary Information

## Figures and Tables

**Table 1:** Genetic diversity, neutral test and statistical results for the test of mismatch distribution based on mitochondrial DNA.

		N	H	Hd	Pi	Tajima’ D	Fu’ F	SSD	Raggedness Index
***N**. **excelsior***	CytB	17	12	0.956	0.013	0.0137	0.125	0.028	0.038
	COI	11	7	0.873	0.017	−1.365	4.669	0.047	0.073
	D-loop	4	4	1.000	0.037	0	1.494	0.125	0.333
Lineage 1	CytB	11	7	0.909	0.003	−0.450	−1.126	0.025	0.062
	COI	5	5	1.000	0.025	−1.362	−1.132	0.093	0.220
	D-loop	3	3	1.000	0.025	0	1.494	0.299	0.667
Lineage 2	CytB	6	5	0.933	0.008	−0.543	0.606	0.086	0.191
	COI	6	3	0.600	0.001	0.160	0.984	0.107	0.204
	D-loop	—	—	—	—	—	—	—	—
***N**. **andersoni***	CytB	73	34	0.967	0.021	−1.127	0.560	0.007	0.004
	COI	57	10	0.850	0.009	0.285	−2.122	0.011	0.015
	D-loop	48	18	0.915	0.031	1.442	3.465	0.028*	0.031*
Lineage 3	CytB	31	18	0.959	0.021	−1.094	1.345	0.697	0.010
	COI	20	5	0.668	0.003	−0.381	4.07	0.056	0.130
	D-loop	15	8	0.790	0.018	−0.269	1.743	0.056	0.076
Lineage 4	CytB	13	6	0.641	0.003	−1.396	2.943	0.081	0.134
	COI	11	2	0.182	0.002	−1.969*	4.128	0.114	0.204
	D-loop	10	2	0.533	0.001	1.302	1.029	0.030	0.289
Lineage 5	CytB	29	10	0.901	0.003	−1.158	−1.264	0.101*	0.045
	COI	26	5	0.662	0.002	3.854	−0.911	0.009	0.962
	D-loop	23	8	0.791	0.004	−0.637	−1.272	0.061	0.213

**Table 2:** Morphological divergence between two genetic lineages.

Groups	BW	HBL	TL	HFL	EL
Holotype of *N. excelsior*		**178** (148?)	**193**	**30**	**23**
*N. excelsior*	67 ± 13.64	133.14 ± 9.788	205.57 ± 12.98	30.71 ± 2.05	22.71 ± 0.95
(n = 7)	(56~95)	(121~145)	(185~221)	(28~33)	(22–24)
Holotype of *N. andersoni*		**164**	**248**	**37**	**26**.**5**
*N. andersoni*	132.71 ± 27.20	170.58 ± 16.38	235.56 ± 16.97	34.42 ± 1.59	22.71 ± 0.95
(n = 31)	(88.1~182)	(136~201)	(203~272)	(30~37)	(24~30)

**Table 3:** Range shifts and environmental conditions in the predicted habitats of the NASC.

Periods	*N. excelsior*	*N. andersoni*	All
**Area** (**10**, **000 Km**^**2**^)			
LIG	50.64	115.15	125.31
LGM	119.57	90.99	110.53
Mid Holocene	112.94	89.62	115.47
Current	124.05	88.80	127.51
Future	66.91	77.72	113.47
**Elevation** (**m**)			
LIG	3096.55 ± 1205.10	2792.11 ± 1457.74	2825.51 ± 1461.47
LGM	2927.75 ± 1499.22	2679.52 ± 1318.84	2813.62 ± 1438.49
Mid Holocene	2931.22 ± 1500.08	2752.24 ± 1307.38	2846.92 ± 1428.91
Current	2937.43 ± 1515.78	2790.75 ± 1351.34	2826.66 ± 1464.56
Future	3424.25 ± 1200.84	2894.67 ± 1257.17	2790.72 ± 1454.77
**MAT** (**Bio1**, **°C**)			
LIG	2.89 ± 6.75	4.12 ± 7.79	4.74 ± 8.30
LGM	2.30 ± 7.60	3.24 ± 6.82	3.19 ± 7.90
Mid Holocene	5.30 ± 7.08	7.20 ± 6.84	6.38 ± 7.33
Current	6.85 ± 7.34	7.34 ± 6.71	7.47 ± 7.41
Future	8.96 ± 6.25	11.70 ± 6.56	11.24 ± 7.12
**MAP** (**Bio12**, **mm**)			
LIG	759.72 ± 326.80	1010.66 ± 525.34	1106.04 ± 602.17
LGM	1464.22 ± 902.56	1058.72 ± 451.10	1192.30 ± 566.45
Mid Holocene	1558.66 ± 893.01	1135.06 ± 491.29	1355.02 ± 643.90
Current	1380.52 ± 744.99	1149.04 ± 471.92	1357 ± 655.79
Future	1555.66 ± 856.37	1180.09 ± 454.79	1327.10 ± 602.12

MAT, mean annual temperature; MAP, mean annual precipitation.

## References

[b1] López-Pujol J., Zhang F. M., Sun H. Q., Ying T. S. & Ge S. Centres of plant endemism in China: places for survival or for speciation? J. Biogeogr. 38, 1267–1280 (2011).

[b2] Wen Z. X. . Multiscale partitioning of small mammal β-diversity provides novel insights into the Quaternary faunal history of Qinghai-Tibetan Plateau and Hengduan mountains. J. Biogeogr. 43, 1412–1424 (2016).

[b3] Ding R. H. The fishes of Sichuan (Sichuan Publishing House of Sciences and Technology, 1994).

[b4] MacKinnon J. & Phillipps K. A field guide to the birds of China (Oxford University Press, 2000).

[b5] Smith A. T. & Xie Y. A guide to the mammals of China (Princeton University Press, 2008).

[b6] Zhu X. L. & Chen Y. R. The fishes of Yunnan (Science Press, 1989–1990).

[b7] Bartlett L. J. . Robustness despite uncertainty: regional climate data reveal the dominant role of humans in explaining global extinctions of Late Quaternary megafauna. Ecography 39, 152–161 (2016).

[b8] Tamma K. & Ramakrishnan U. Higher speciation and lower extinction rates influence mammal diversity gradients in Asia. BMC Evol. Biol. 15, 11 (2015). 25648944 10.1186/s12862-015-0289-1 PMC4333168

[b9] He K., Hu N. Q., Chen X., Li J. T. & Jiang X. L. Interglacial refugia preserved high genetic diversity of the Chinese mole shrew in the mountains of southwest China. Heredity 116, 23–32 (2016). 26286667 10.1038/hdy.2015.62 PMC4675870

[b10] Qu Y. H., Lei F. M., Zhang R. Y. & Lu X. Comparative phylogeography of five avian species: implications for Pleistocene evolutionary history in the Qinghai-Tibetan plateau. Mol. Ecol. 19, 338–351 (2010). 20002586 10.1111/j.1365-294X.2009.04445.x

[b11] Wang Z. W. . Climatic factors drive population divergence and demography: insights based on the phylogeography of a riparian plant species endemic to the Hengduan mountains and adjacent regions. PLoS One 10, e0145014 (2015). 26689776 10.1371/journal.pone.0145014 PMC4687034

[b12] Bekken D., Schepartz L. A., Miller-Antonio S., Yamei H. & Weiwen H. Taxonomic abundance at Panxian Dadong, a Middle Pleistocene cave in south China. Asian Perspect. 43, 333–359 (2004).

[b13] Li B. S. . Paleoclimate change recorded in the red earth and brown-yellow sediment of Late Quaternary for northeastern part of Guangdong Province, south to the Nanling mountains, China. Chin. Sci. Bull. 53, 3866–3875 (2008).

[b14] Liu W., Wu X. Z., Pei S. W., Wu X. J. & Norton C. J. Huanglong cave: a Late Pleistocene human fossil site in Hubei Province, China. Quat. Int. 211, 29–41 (2010).

[b15] Tong H. W. & Wu X. Z. *Stephanorhinus kirchbergensis* (Rhinocerotidae, Mammalia) from the Rhino Cave in Shennongjia, Hubei. Chin. Sci. Bull. 55, 1157–1168 (2010).

[b16] Faith J. T. & Surovell T. A. Synchronous extinction of north America’s Pleistocene mammals. Proc. Natl. Acad. Sci. USA 106, 20641–20645 (2009). 19934040 10.1073/pnas.0908153106 PMC2791611

[b17] Hu Y. B., Qi D. W., Wang H. J. & Wei F. W. Genetic evidence of recent population contraction in the southernmost population of giant pandas. Genetica 138, 1297–1306 (2010). 21120682 10.1007/s10709-010-9532-2

[b18] Li R. Q. . Climate change-induced decline in bamboo habitats and species diversity: implications for giant panda conservation. Divers. Distrib. 21, 379–391 (2015).

[b19] Shen G. Z. . Climate change challenges the current conservation strategy for the giant panda. Biol. Conserv. 190, 43–50 (2015).

[b20] Swaisgood R. R., Wei F. W., Wildt D. E., Kouba A. J. & Zhang Z. J. Giant panda conservation science: how far we have come. Biol. Lett. 6, 143–145 (2010). 19864275 10.1098/rsbl.2009.0786 PMC2865067

[b21] Guthrie R. D. New carbon dates link climatic change with human colonization and Pleistocene extinctions. Nature 441, 207–209 (2006). 16688174 10.1038/nature04604

[b22] Li X. H. . Human impact and climate cooling caused range contraction of large mammals in China over the past two millennia. Ecography 38, 74–82 (2015).

[b23] Craw D., Upton P., Burridge C. P., Wallis G. P. & Waters J. M. Rapid biological speciation driven by tectonic evolution in New Zealand. Nat. Geosci. 9, 140–144 (2016).

[b24] Ricklefs R. E. Global diversification rates of passerine birds. Proc. R. Soc. B. 270, 2285–2291 (2003). 10.1098/rspb.2003.2489 PMC1691496 14613616

[b25] Lorenzen E. D. . Species-specific responses of Late Quaternary megafauna to climate and humans. Nature 479, 359–364 (2011). 22048313 10.1038/nature10574 PMC4070744

[b26] Mapelli F. J., Mora M. S., Mirol P. M. & Kittlein M. J. Effects of Quaternary climatic changes on the phylogeography and historical demography of the subterranean rodent *Ctenomys porteousi*. J. Zool. 286, 48–57 (2012).

[b27] Domingo L. . Late Neogene and early Quaternary paleoenvironmental and paleoclimatic conditions in southwestern Europe: isotopic analyses on mammalian taxa. PLoS One 8, e63739 (2013). 23717470 10.1371/journal.pone.0063739 PMC3662777

[b28] Blois J. L., McGuire J. L. & Hadly E. A. Small mammal diversity loss in response to late-Pleistocene climatic change. Nature 465, 771–774 (2010). 20495547 10.1038/nature09077

[b29] Prost S. . Losing ground: past history and future fate of Arctic small mammals in a changing climate. Glob. Change Biol. 19, 1854–1864 (2013). 10.1111/gcb.12157 23505210

[b30] Hernández F. M. Bioclimatic discriminant capacity of terrestrial mammal faunas. Glob. Ecol. Biogeogr. 10, 189–204 (2001).

[b31] Leach K., Kelly R., Cameron A., Montgomery W. I. & Reid N. Expertly validated models and phylogenetically-controlled analysis suggests responses to climate change are related to species traits in the order Lagomorpha. PLoS One 10, e0122267 (2015). 25874407 10.1371/journal.pone.0122267 PMC4398435

[b32] McCain C. M. & King S. R. Body size and activity times mediate mammalian responses to climate change. Glob. Change Biol. 20, 1760–1769 (2014). 10.1111/gcb.12499 24449019

[b33] Yue H. . A mitogenome of the Chevrier’s field mouse (*Apodemus chevrieri*) and genetic variations inferred from the cytochrome b gene. DNA Cell Biol. 31, 460–469 (2012). 21870961 10.1089/dna.2011.1301

[b34] Fan Z. X., Liu S. Y., Liu Y., Zhang X. Y. & Yue B. S. How Quaternary geologic and climatic events in the southeastern margin of the Tibetan Plateau influence the genetic structure of small mammals: inferences from phylogeography of two rodents, *Neodon irene* and *Apodemus latronum*. Genetica 139, 339–351 (2011). 21298554 10.1007/s10709-011-9553-5

[b35] Suzuki Y., Tomozawa M., Koizumi Y., Tsuchiya K. & Suzuki H. Estimating the molecular evolutionary rates of mitochondrial genes referring to Quaternary ice age events with inferred population expansions and dispersals in Japanese *Apodemus*. BMC Evol. Biol. 15, 187 (2015). 26373638 10.1186/s12862-015-0463-5 PMC4571126

[b36] Lu L. . Molecular phylogeny and the underestimated species diversity of the endemic white-bellied rat (Rodentia: Muridae:*Niviventer*) in Southeast Asia and China. Zool. Scr. 44, 475–494 (2015).

[b37] Musser G. G. & Carleton M. D. Mammal species of the world: a taxonomic and geographic reference (eds Wilson D. E. & Reeder D. A. M.) 894–1531 (Johns Hopkins University Press, 2005).

[b38] Balakirev A. E., Abramov A. V. & Rozhnov V. V. Taxonomic revision of *Niviventer* (Rodentia, Muridae) from Vietnam: a morphological and molecular approach. Russ. J. Theriol. 10, 1–26 (2011).

[b39] Balakirev A. E. & Rozhnov V. V. Phylogenic relationships and species composition in the genus *Niviventer* (Rodentia, Muridae) based on studies of the cytochrome b gene of mtDNA. Moscow Univ. Biol. Sci. Bull. 65, 170–173 (2010).

[b40] Jing M. D., Yu H. T., Wu S. H., Wang W. & Zheng X. G. Phylogenetic relationships in genus *Niviventer* (Rodentia: Muridae) in China inferred from complete mitochondrial cytochrome b gene. Mol. Phylogenet. Evol. 44, 521–529 (2007). 17531508 10.1016/j.ympev.2007.04.003

[b41] Chen W. C., Sun Z. Y., Liu Y., Yue B. S. & Liu S. Y. The complete mitochondrial genome of the large white-bellied rat, *Niviventer excelsior* (Rodentia: Muridae). Mitochondrial DNA 23, 363–365 (2012). 22775427 10.3109/19401736.2012.696627

[b42] Chen W. C., Yue B. S. & Liu S. Y. Complex terrain and climatic oscillations in Hengduan mountains influence geographical division of the large white-bellied rat (*Niviventer excelsior*). Sichuan. J. Zool. 29, 346–351 (2010).

[b43] Chen W. C. . Phylogeography of the large white-bellied rat *Niviventer excelsior* suggests the influence of Pleistocene glaciations in the Hengduan mountains. Zool. Sci. 27, 487–493 (2010). 20528155 10.2108/zsj.27.487

[b44] He K. & Jiang X. L. Mitochondrial phylogeny reveals cryptic genetic diversity in the genus *Niviventer* (Rodentia, Muroidea). Mitochondrial DNA 26, 48–55 (2015). 24021005 10.3109/19401736.2013.823167

[b45] Augustin L. . Eight glacial cycles from an Antarctic ice core. Nature 429, 623–628 (2004). 15190344 10.1038/nature02599

[b46] Provan J. & Bennett K. D. Phylogeographic insights into cryptic glacial refugia. Trends Ecol. Evol. 23, 564–571 (2008). 18722689 10.1016/j.tree.2008.06.010

[b47] Lanier H. C., Gunderson A. M., Weksler M., Fedorov V. B. & Olson L. E. Comparative phylogeography highlights the double-edged sword of climate change faced by arctic- and alpine-adapted mammals. PLoS One 10, e0118396 (2015). 25734275 10.1371/journal.pone.0118396 PMC4348485

[b48] Stewart J. R., Lister A. M., Barnes I. & Dalen L. Refugia revisited: individualistic responses of species in space and time. Proc. R. Soc. B. Biol. Sci. 277, 661–671 (2010). 10.1098/rspb.2009.1272 PMC2842738 19864280

[b49] Millar C. I. & Westfall R. D. Distribution and climatic relationships of the American pika (*Ochotona princeps*) in the Sierra Nevada and Western Great Basin, USA; Periglacial landforms as refugia in warming climates. Arct. Antarct. Alp. Res. 42, 493–496 (2010).

[b50] Beever E. A., Ray C., Wilkening J. L., Brussard P. F. & Mote P. W. Contemporary climate change alters the pace and drivers of extinction. Glob. Change Biol. 17, 2054–2070 (2011).

[b51] Galbreath K. E., Hafner D. J. & Zamudio K. R. When cold is better: climate-driven elevation shifts yield complex patterns of diversification and demography in an alpine specialist American pika (*Ochotona princeps*). Evolution 63, 2848–2863 (2009). 19663994 10.1111/j.1558-5646.2009.00803.x

[b52] Kohli B. A., Fedorov V. B., Waltari E., Cook J. A. & Riddle B. Phylogeography of a Holarctic rodent (*Myodes rutilus*): testing high-latitude biogeographical hypotheses and the dynamics of range shifts. J. Biogeogr. 42, 377–389 (2015).

[b53] Gür H. The effects of the Late Quaternary glacial-interglacial cycles on Anatolian ground squirrels: range expansion during the glacial periods? Biol. J. Linn. Soc. 109, 19–32 (2013).

[b54] Zheng S. H. Quaternary rodents of Sichuan-Guizhou area, China (Science Press, 1993).

[b55] Jin C. Z., Zheng J. J., Wang Y. & Xu Q. Q. The stratigraphic distribution and zoogeography of the early Pleistocene mammalian fauna from south China. Acta Anthrop. Sinica 27, 304–317 (2008).

[b56] Schepartz L. A., Stoutamire S. & Bekken D. A. *Stegodon orientalis* from Panxian Dadong, a Middle Pleistocene archaeological site in Guizhou, South China: taphonomy, population structure and evidence for human interactions. Quat. Int. 126–128, 271–282 (2005).

[b57] Librado P. & Rozas J. DnaSP v5: a software for comprehensive analysis of DNA polymorphism data. Bioinformatics 25, 1451–1452 (2009). 19346325 10.1093/bioinformatics/btp187

[b58] Thomas O. The Duke of Bedford’s zoological exploration of Eastern Asia; xiv. On Mammals from Southern Shensi, Central China. Proc. Zool. Soc. London 81, 687–696 (1911).

[b59] Rowe K. C., Heske E. J. & Paige K. N. Comparative phylogeography of eastern chipmunks and white-footed mice in relation to the individualistic nature of species. Mol. Ecol. 15, 4003–4020 (2006). 17054499 10.1111/j.1365-294X.2006.03063.x

[b60] Horikawa K. . Pliocene cooling enhanced by flow of low-salinity Bering Sea water to the Arctic Ocean. Nat. Commun. 6, 7587 (2015). 26119338 10.1038/ncomms8587 PMC4491831

[b61] Head M. J. & Gibbard P. L. Early-Middle Pleistocene transitions: an overview and recommendation for the defining boundary. Geol. Soc. London, Spec. Publ. 247, 1–18 (2005).

[b62] Berger W. H., Yasuda M. K., Bickert T., Wefer G. & Takayama T. Quaternary time scale for the Ontong Java Plateau: Milankovitch template for Ocean Drilling Program Site 806. Geology 22, 463–467 (1994).

[b63] Ruddiman W. F., Raymo M. E., Martinson D. G., Clement B. M. & Backman J. Pleistocene evolution: Northern hemisphere ice sheets and North Atlantic Ocean. Paleoceanography 4, 353–412 (1989).

[b64] Li W. Y. Vegetation and enviroment of east China in Quaternary cold stage. Acta Geolog. Sinica 42, 299–307 (1987).

[b65] Shi Y. F., Zheng B. X. & Yao T. D. Glaciers and environments during the Last Glacial Maximum (LGM) on the Tibetan Plateau. J. Glaciol. Geocryol. 19, 97–113 (1997).

[b66] Zhao S. C. . Whole-genome sequencing of giant pandas provides insights into demographic history and local adaptation. Nat. Genet. 45, 67–71 (2013). 23242367 10.1038/ng.2494

[b67] Zhou X. M. . Whole-genome sequencing of the snub-nosed monkey provides insights into folivory and evolutionary history. Nat. Genet. 46, 1303–1310 (2014). 25362486 10.1038/ng.3137

[b68] Yang S. L. & Ding Z. L. A 249 kyr stack of eight loess grain size records from northern China documenting millennial-scale climate variability. Geochem. Geophys. Geosyst. 15, 798–814 (2014).

[b69] Yi C. L. . Advances in numerical dating of Quaternary glaciations in China. Z. Geomorphol. 51, 153–175 (2007).

[b70] Bacon A. M. . Records of murine rodents (Mammalia, Rodentia) in the Pleistocene localities of Tan Vinh and Ma U’Oi (Northern Vietnam) and their implications to past distribution. Ann. Paléon. 92, 367–383 (2006).

[b71] Jin C. Z. . Preliminary report on the 2002 excavation of Jinpendong site at Wuhu, Anhui Province. Acta Anthrop. Sinica 23, 281–291 (2004).

[b72] Wang Y. . Murid rodents of the newly discovered *Gigantopithecus* fauna from the Sanhe Cave, Chongzuo, Guangxi, South China. Acta Anthrop. Sinica 28, 73–87 (2009).

[b73] Chen R. . Mid- to late-Holocene East Asian summer monsoon variability recorded in lacustrine sediments from Jingpo Lake, Northeastern China. Holocene 25, 454–468 (2015).

[b74] Zhao Y., Yu Z. C., Chen F. H., Zhang J. W. & Yang B. Vegetation response to Holocene climate change in monsoon-influenced region of China. Earth-Sci. Rev. 97, 242–256 (2009).

[b75] Charlotte G. C., Richard. J., Peter G. L., Melanie J. L. & Zhang E. L. New insights on late Quaternary Asian palaeomonsoon variability and the timing of the Last Glacial Maximum in southwestern China. Quat. Sci. Rev. 30, 808–820 (2011).

[b76] Huang J. H. . Identifying hotspots of endemic woody seed plant diversity in China. Divers. Distrib. 18, 673–688 (2012).

[b77] Vrba E. S. Mammals as a key to evolutionary theory. J. Mammal. 73, 1–28 (1992).

[b78] Cantalapiedra J. L., Hernández Fernández M. & Morales J. Biomic specialization and speciation rates in ruminants (*Cetartiodactyla*, Mammalia): a test of the resource-use hypothesis at the global scale. PLoS One 6, e28749 (2011). 22174888 10.1371/journal.pone.0028749 PMC3236210

[b79] Schneider von Deimling T., Ganopolski A., Held H. & Rahmstorf S. How cold was the Last Glacial Maximum? Geophys. Res. Lett. 33, L14709 (2006).

[b80] Cook C. G., Jones R. T., Langdon P. G., Leng M. J. & Zhang E. New insights on Late Quaternary Asian palaeomonsoon variability and the timing of the Last Glacial Maximum in southwestern China. Quat. Sci. Rev. 30, 808–820 (2011).

[b81] Liang F. Dynastic data of China’s households, cultivated land and land taxation (Shanghai People’s Press, 1980).

[b82] White T. E. A method of calculating the dietary percentage of various food animals utilized by aboriginal peoples. Am. Antiq. 18, 396–398 (1953).

[b83] Musser G. G. & Chiu S. Notes on taxonomy of *Rattus andersoni* and *R. excelsior*, murids endemic to western China. J. Mammal. 60, 581–592 (1979).

[b84] Irwin D. M., Kocher T. D. & Wilson A. C. Evolution of the cytochrome b gene of mammals. J. Mol. Evol. 32, 128–144 (1991). 1901092 10.1007/BF02515385

[b85] Pages M. . Revisiting the taxonomy of the Rattini tribe: a phylogeny-based delimitation of species boundaries. BMC Evol. Biol. 10, 184 (2010). 20565819 10.1186/1471-2148-10-184 PMC2906473

[b86] Poux C. & Douzery E. J. Primate phylogeny, evolutionary rate variations, and divergence times: a contribution from the nuclear gene IRBP. Am. J. Phys. Anthropol. 124, 1–16 (2004). 15085543 10.1002/ajpa.10322

[b87] Robins J. H., Hingston M., Matisoo-Smith E. & Ross H. A. Identifying *Rattus* species using mitochondrial DNA. Mol. Ecol. Notes 7, 717–729 (2007).

[b88] Li J. . DNA barcoding of Murinae (Rodentia: Muridae) and Arvicolinae (Rodentia: Cricetidae) distributed in China. Mol. Ecol. Resour. 15, 153–167 (2015). 24838015 10.1111/1755-0998.12279

[b89] Thompson J. D., Gibson T. J. & Higgins D. G. Current protocols in bioinformatics (John Wiley & Sons, Inc., 2002).

[b90] Tamura K., Stecher G., Peterson D., Filipski A. & Kumar S. MEGA6: molecular evolutionary genetics analysis version 6.0. Mol. Biol. Evol. 30, 2725–2729 (2013). 24132122 10.1093/molbev/mst197 PMC3840312

[b91] Keane T. M., Creevey C. J., Pentony M. M., Naughton T. J. & McLnerney J. O. Assessment of methods for amino acid matrix selection and their use on empirical data shows that ad hoc assumptions for choice of matrix are not justified. BMC Evol. Biol. 6, 29 (2006). 16563161 10.1186/1471-2148-6-29 PMC1435933

[b92] Ronquist F. . MrBayes 3.2: efficient Bayesian phylogenetic inference and model choice across a large model space. Syst. Biol. 61, 539–542 (2012). 22357727 10.1093/sysbio/sys029 PMC3329765

[b93] Rambaut A. & Drummond A. J. Tracer v1.5 http://beast.bio.ed.ac.uk/Tracer (2007).

[b94] Bandelt H. J., Forster P. & Rohl A. Median-joining networks for inferring intraspecific phylogenies. Mol. Biol. Evol. 16, 37–48 (1999). 10331250 10.1093/oxfordjournals.molbev.a026036

[b95] Polzin T. & Daneshmand S. V. On Steiner trees and minimum spanning trees in hypergraphs. Oper. Res. Lett. 31, 12–20 (2003).

[b96] Tajima F. Statistical method for testing the neutral mutation hypothesis by DNA polymorphism. Genetics 123, 585–595 (1989). 2513255 10.1093/genetics/123.3.585 PMC1203831

[b97] Fu Y. X. Statistical tests of neutrality of mutations against population growth, hitchhiking and background selection. Genetics 147, 915–925 (1997). 9335623 10.1093/genetics/147.2.915 PMC1208208

[b98] Excoffier L., Laval G. & Schneider S. Arlequin (version 3.0): an integrated software package for population genetics data analysis. Evol. Bioinf. Online 1, 47–50 (2005). PMC2658868 19325852

[b99] Drummond A. J., Rambaut A., Shapiro B. & Pybus O. G. Bayesian coalescent inference of past population dynamics from molecular sequences. Mol. Biol. Evol. 22, 1185–1192 (2005). 15703244 10.1093/molbev/msi103

[b100] Avise J. C. The history and purview of phylogeography: a personal reflection. Mol. Ecol. 7, 371–379 (1998).

[b101] Pesole G., Gissi C., De Chirico A. & Saccone C. Nucleotide substitution rate of mammalian mitochondrial genomes. J. Mol. Evol. 48, 427–434 (1999). 10079281 10.1007/pl00006487

[b102] Barry J. C. . Faunal and environmental change in the late Miocene Siwaliks of northern Pakistan. Paleobiology 28, 1–71 (2002).

[b103] Johnson N. M., Stix J., Tauxe L., Cerveny P. F. & Tahirkheli R. A. K. Paleomagnetic chronology, fluvial processes, and tectonic implications of the Siwalik deposits near Chinji Village, Pakistan. J. Geol. 93, 27–40 (1985).

[b104] Aguilar J. & Michaux J. The beginning of the age of Murinae (Mammalia: Rodentia) in southern France. Acta Zool. Crac. 39, 35–45 (1996).

[b105] Drummond A. J. & Rambaut A. BEAST: bayesian evolutionary analysis by sampling trees. BMC Evol. Biol. 7, 214 (2007). 17996036 10.1186/1471-2148-7-214 PMC2247476

[b106] Rambaut A. & Drummond A. J. TreeAnnotator 2.1.2 http://beast.Bio.Ed.Ac.Uk/TreeAnnotator (2007).

[b107] Phillips S. J. & Dudík M. Modeling of species distributions with Maxent: new extensions and a comprehensive evaluation. Ecography 31, 161–175 (2008).

[b108] Pearson R. G., Raxworthy C. J., Nakamura M. & Peterson A. T. Predicting species distributions from small numbers of occurrence records: a test case using cryptic geckos in Madagascar. J Biogeogr. 34, 102–117 (2007).

