# Supplementary material for: An endemic rat species complex is evidence of moderate environmental changes in the terrestrial biodiversity centre of China through the late Quaternary
Source: Sci Rep. 2017 Apr 10;7:46127. doi: 10.1038/srep46127 (PMC5385558; doi:10.1038/srep46127)
Supplement: Supplementary Information [file srep46127-s1.pdf]

An endemic rat species complex is evidence of moderate environmental changes in the terrestrial biodiversity centre of China through the late Quaternary

Deyan Ge[1], Liang Lu[2], Jilong Cheng[1,3], Lin Xia[1], Yongbin Chang[1], Zhixin Wen[1], Xue Lv[1,3], Yuanbao Du[1,3], Qiyong Liu[2], Qisen Yang[1]

[1] Key Laboratory of Zoological Systematics and Evolution, Institute of Zoology, Chinese Academy of Sciences, Beijing 100101,China. [2] State Key Laboratory for Infectious Diseases Prevention and Control, National Institute for Communicable Disease Control and Prevention, Chinese Center for Disease Control and Prevention, Beijing, 102206, China. [3]University of Chinese Academy of Sciences, Beijing, 100041. China. Correspondence and requests for materials should be addressed to Q. S. Y. (email: yangqs@ioz.ac.cn). Deyan Ge and Liang Lu contributed equally to the present study.

Table S1. Fossil records of the NACS. The relative sample abundance of fossils from each sites were counted using Minimum Number of Individuals. The Quaternary rodents of Sichuan and Guizhou supports the recognition of five periods: Taimiao (2.6-1.8 Mya), Tianqiao (1.8-1.1 Mya), Yanjingou I (1.1-0.45 Mya), Geleshan (0.45-0.13 Mya), and Yanjingou II (0.13-0.01 Mya) [1]. Occurences of the NASC were dated in the later four periods, classified as 1 to 4 in fourth volume.

Number	Museum	Period 1	Period 2	Original Identification	Location of specimen (Location codes in Figure 1)	Sample abundance	Elevation	Longitude	Latitude	References
1	IVPP	Pleistocene	1	*N. andersoni*	China,Chongqing, Wushan, Longgupo	104	1200	110.00	31.10	1
2		Pleistocene	2	*N. andersoni*	China, Guizhou, Panxian, Da cave	Unknow	1655	104.73	25.63	2
3	IVPP	Pleistocene	3	*N. andersoni*	China, Guizhou, Puding, Baiyanjiao Cave	5	1280	105.68	25.25	1
4	IVPP	Pleistocene	3	*N. andersoni*	China, Guizhou, Tongzhi, Chuan cave,	1	1340	105.75	26.30	1
5	IVPP	Pleistocene	3	*N. andersoni*	China, Yanhui Cave	8	1325	106.73	28.20	1
6	IVPP	Pleistocene	3	*N. andersoni*	China, Guizhou, Tongzhi, Wazuwan Cave	6	1340	106.73	28.21	1
7	IVPP	Pleistocene	3	*N. andersoni*	China, Guizhou, Tongzhi, Tianmen Cave	55	1000	106.80	28.16	1
8		Pleistocene	3	*N. andersoni*	China, Shanxi, Longshan	Unknow		106.86	34.91	3
9		Pleistocene	3	*N. andersoni*	China, Shanxi,Ankang	Unknow		109.02	32.70	3
10		Pleistocene	3	*N. andersoni*	China, Shanxi, Xishui Cave	Unknow	1651	109.40	34.40	3
11	IVPP	Pleistocene	3	*N. andersoni*	China, Chongqing, Baotan Temple	47	820	109.65	30.85	1
12		Pleistocene	4	*N. andersoni*	Vietnam, Hoa Binh province, Northern, Ma U'Oi	Unknow	2000	105.26	20.61	4
13	IVPP	Pleistocene	4	*N. andersoni*	China, Chongqing, Wanxian, Pingbai	110	880-920	108.35	30.83	1
14	IVPP	Pleistocene	1	*N. andersoni*	China, Guangxi, Chongzuo, Boyue Mountain	3	206	107.50	22.31	5
15		Pleistocene	4	*N. andersoni*	China, Hunan, Yanger Cave	6	319	109.26	28.50	6
16		Pleistocene	4	*N. andersoni*	China, Jiangxi, Pingxiang		235	113.81	27.78	7
17	IVPP	Pleistocene	1	*N. excelsior*	China, Guizhou, Weining, Tianqiaoniexi	Unknow		104.12	26.48	
18		Pleistocene	3	*N. excelsior*	China, Guizhou, Bijie, Mawokou Cave	Unknow	1450-700	105.36	27.71	8
19		Pleistocene	3	*N. excelsior*	China,Anhui, Wuhu, Jinpeng Cave	Unknow		118.37	31.21	9

Table S2 Collected information from molecular voucher specimens and their GenBank accession numbers.

Number	Museum	CytB	COI	D-loop	IRBP	Location	Elevation	Longitude	Latitude	References
2		EF053003*				Qinglin,Meixian,Shanxi,China		107.00	34.00	10
3		EF053001*				YuLong Mount, Lijiang, Yunnan, China		100.00	27.00	10
4047		EF053002*				Mount Ailao,Jingdong,Yunnan,China		101.01	24.53	10
5029		EF053007*				Ailao Mount, Jingdong, Yunnan, China		101.01	24.53	10
5030		EF053004*				Ailao Mount, Jingdong, Yunnan, China		101.01	24.53	10
5043		EF053005*				Ailao Mount, Jingdong, Yunnan, China		101.01	24.53	10
5070		EF053006*				Ailao Mount, Jingdong, Yunnan, China		101.01	24.53	10
14JD032	IOZCAS	KU531733	KU531758	*KU531803*	KU531840	Jiangda, Tibet,China	3468	98.26	32.53	This study
AZ074	IOZCAS	KF740007	KU531783		KU531841	Luojishan, Puge, Sichuan, China	3400	102.20	27.40	This study
BMAYB109	IOZCAS	KU531734	KU531759	*KU531804*	KU531842	Baimang Snow Mountain, Deqin, Yunnan, China	3100	99.37	27.63	This study
BMAYB132	IOZCAS	KU531735	KU531760	*KU531805*	KU531843	Baimang Snow Mountain, Deqin, Yunnan, China	3100	99.37	27.63	This study
BMAYC471	IOZCAS	KU531736	KU531761			Baimang Snow Mountain, Deqin, Yunnan, China	3100	99.37	27.63	This study
BMAYC501	IOZCAS	KU531737	KU531762	*KU531806*	KU531844	Baimang Snow Mountain, Deqin, Yunnan, China	3100	99.37	27.63	This study
BMAYC529	IOZCAS	KF739992	KU531763	*KU531807*	KU531845	Baimang Snow Mountain, Deqin, Yunnan, China	3100	99.37	27.63	This study
BMAYD491	IOZCAS	KF739993	KF739982	KF739916	KF769319	Baimang Snow Mountain, Deqin, Yunnan, China	3100	99.37	27.63	This study
BMAYD516	IOZCAS	KF739995	KU531764	*KU531808*		Baimang Snow Mountain, Deqin, Yunnan, China	3100	99.37	27.63	This study
BMAYD526	IOZCAS	KF739996	KU531765	*KU531809*	KU531846	Baimang Snow Mountain, Deqin, Yunnan, China	3100	99.37	27.63	This study
BMBZA137	IOZCAS	KU531738	KU531766	*KU531810*	KU531847	Baimang Snow Mountain, Deqin, Yunnan, China	3400	99.37	27.63	This study
BMBZC449	IOZCAS	KF740029	KU531767	*KU531811*	KU531848	Baimang Snow Mountain, Deqin, Yunnan, China	3400	99.37	27.63	This study
BMBZD446	IOZCAS	KF740030	KU531768	*KU531812*	KU531849	Baimang Snow Mountain, Deqin, Yunnan, China	3400	99.37	27.63	This study
BT003		HM037045				Batang, Sichuan, China		99.00	30.00	11
CWL52		HM037044				Chawalong, Xizang, China		98.20	28.46	11
DJ20140069	IOZCAS	KU531740	KU531769	*KU531813*	KU531851	Dongjiu, Seji Moutain, Tibet, China	2600	94.77	29.70	This study

Number	Museum	CytB	COI	D-loop	IRBP	Location	Elevation	Longitude	Latitude	References
DJ20140079	IOZCAS	KU531741	KU531770	*KU531814*	KU531852	Dongjiu, Seji Moutain, Tibet, China	2600	94.77	29.70	This study
f110	SCU		KF999172			Fengtongzai National Reserve, Sichuan, China	1555	102.88	30.57	12
f14	SCU		KF999171			Fengtongzai National Reserve, Sichuan, China	1620	102.88	30.59	12
GGCA20266	IOZCAS	KF740031	*KP745896	*KP746078	KF978173	Gongga Mountain, Luding, Sichuan, China	3200	101.50	29.50	This study
GGDB20045	IOZCAS	KU531739	*KP745945	*KP746082	*KP746298	Gongga Mountain, Luding, Sichuan, China	1600	101.50	29.50	This study
GGDB20064	IOZCAS	KF740072	*KP746060	*KP746084	*KP746388	Gongga Mountain, Luding, Sichuan, China	1600	101.50	29.50	This study
GGHA10233	IOZCAS	KF740093	*KP745958	*KP746083	KF978174	Gongga Mountain, Luding, Sichuan, China	2800	101.50	29.50	This study
GGHA20338	IOZCAS	*KP754616	*KP745966	*KP746085	*KP746317	Gongga Mountain, Luding, Sichuan, China	2800	101.50	29.50	This study
GGPB20138	IOZCAS	*KP754637	*KP746006	*KP746087	*KP746347	Gongga Mountain, Luding, Sichuan, China	2400	101.50	29.50	This study
GGPB20149	IOZCAS	*KP754638	*KP746009	*KP746086	*KP746349	Gongga Mountain, Luding, Sichuan, China	2400	101.50	29.50	This study
GGPB20172	IOZCAS	KF740205		*KU531822*		Gongga Mountain, Luding, Sichuan, China	2400	101.50	29.50	This study
GGPB20183	IOZCAS	KF740206	KP746013	KP746081	KF978177	Gongga Mountain, Luding, Sichuan, China	2400	101.50	29.50	This study
GGYA10161	IOZCAS	KF740231				Gongga Mountain, Luding, Sichuan, China	2000	101.50	29.50	This study
GGYB20064	IOZCAS	*KP754658	*KP746060	*KP746084	*KP746388	Gongga Mountain, Luding, Sichuan, China	2000	101.50	29.50	This study
GGYB20077	IOZCAS	KF740257	*KP746064	*KP746076	KF978141	Gongga Mountain, Luding, Sichuan, China	2000	101.50	29.50	This study
GLGS065		EF053008				Gaoligong, Lushui, Yunnan, China		103.20	25.80	10
GLGS066		EF053009				Gaoligong, Lushui, Yunnan, China		103.20	25.80	10
JJSA019		HM037049				Jiajinshan, Sichuan, China		102.42	30.33	11
JJSA020		HM037050				Jiajinshan, Sichuan, China		102.42	30.33	11
JJSA608		HM037048				Jiajinshan, Sichuan, China		102.42	30.33	11
JL003		HM037047				Jiulong, Sichuan,China		101.25	28.74	11
LZA001	IOZCAS	KU531743	KU531784			Luojishan, Puge, Sichuan, China	3000	102.20	27.40	This study
m21	SCU		KF999096			Dafengding National Reserve,Mabian, Sichuan,China	2775	103.29	28.54	12
m22	SCU		KF999097			Dafengding National Reserve,Mabian, Sichuan,China	2775	103.29	28.54	12

Number	Museum	CytB	COI	D-loop	IRBP	Location	Elevation	Longitude	Latitude	References
m34	SCU		KF999098			Dafengding National Reserve,Mabian, Sichuan,China	2659	103.29	28.54	12
m35	SCU		KF999099			Dafengding National Reserve,Mabian, Sichuan,China	2659	103.29	28.54	12
m9	SCU		KF999095			Dafengding National Reserve,Mabian, Sichuan,China	2761	103.29	28.54	12
MGSCH115	IOZCAS	KU531744	KU531785	*KU531821*	KU531862	Dafengding National Reserve,Mabian, Sichuan,China	2500	103.29	28.54	This study
MSWC164		DQ191482				Shimian, Sichuan, China		102.38	29.25	13
SCE032		GU479937*				Jiulong, Sichuan, China		101.25	28.74	11
SCSD003		GU479936*				Danba, Sichuan, China		101.90	30.88	11
SCSH1		GU479938*				Luhuo, Sichuan, China		101.65	31.38	11
SCSH3		GU479939*				Luhuo, Sichuan, China		101.65	31.38	11
SCSK3		GU479940*				Kangding, Sichuan, China		102.03	32.05	11
SCSL007		GU479941*				Erlangshan, Sichuan, China		102.33	29.97	11
SNJPQ008	IOZCAS	KF740209	KU531786	*KU531823*	KU531863	Shengnongjia, Hubei, China	1400	110.68	31.74	This study
TJHHHC370	IOZCAS	KF740115	KF739984	KF739913	KF769343	Tangjiahe, Qingchuan, Sichuan, China	2200	104.74	32.60	This study
TJHHHC371	IOZCAS	KF740116	KU531771	*KU531815*	KU531853	Tangjiahe, Qingchuan, Sichuan, China	2200	104.74	32.60	This study
TJHHHC375	IOZCAS	KF740117	KU531772	*KU531816*	KU531854	Tangjiahe, Qingchuan, Sichuan, China	2200	104.74	32.60	This study
TJHHHC378	IOZCAS	KF740119	KU531773	*KU531817*	KU531855	Tangjiahe, Qingchuan, Sichuan, China	2200	104.74	32.60	This study
TJHHHD366	IOZCAS	KF740121	KU531774	*KU531818*	KU531856	Tangjiahe, Qingchuan, Sichuan, China	2200	104.74	32.60	This study
TJHHYC129	IOZCAS	KF740139	KU531775	*KU531819*	KU531857	Tangjiahe, Qingchuan, Sichuan, China	1500	104.74	32.60	This study
TJHYCC396	IOZCAS	KF740264	KU531799	*KU531836*	KU531876	Tangjiahe, Qingchuan, Sichuan, China	2550	104.74	32.60	This study
TJHYCD384	IOZCAS	KU531757	KU531801	*KU531838*	KU531878	Tangjiahe, Qingchuan, Sichuan, China	2550	104.74	32.60	This study
TJHYCD404	IOZCAS	KF740265	KU531800	*KU531837*	KU531877	Tangjiahe, Qingchuan, Sichuan, China	2550	104.74	32.60	This study
TJHYCD413	IOZCAS	KF740266	KF739985	KF739914	KF769370	Tangjiahe, Qingchuan, Sichuan, China	2550	104.74	32.60	This study
WL14015	IOZCAS	KU531745	KU531787	*KU531824*	KU531864	Wolong Nature reserve, Wenchuan, Sichuan, China	1930	102.90	30.80	This study
WL14022	IOZCAS	KU531746	KU531788	*KU531825*	KU531865	Wolong Nature reserve, Wenchuan, Sichuan, China	1930	102.90	30.80	This study

Number	Museum	CytB	COI	D-loop	IRBP	Location	Elevation	Longitude	Latitude	References
WL140285	IOZCAS	KU531747	KU531789	*KU531826*	KU531866	Wolong Nature reserve, Wenchuan, Sichuan, China	2200	102.90	30.80	This study
WL14288	IOZCAS	KU531748	KU531790	*KU531827*	KU531867	Wolong Nature reserve, Wenchuan, Sichuan, China	2200	102.90	30.80	This study
WL14315	IOZCAS	KU531749	KU531791	*KU531828*	KU531868	Wolong Nature reserve, Wenchuan, Sichuan, China	2200	102.90	30.80	This study
WL15005	IOZCAS	KU531750	KU531792	*KU531829*	KU531869	Wolong Nature reserve, Wenchuan, Sichuan, China	1700	102.90	30.80	This study
WL15074	IOZCAS	KU531751	KU531793	*KU531830*	KU531870	Wolong Nature reserve, Wenchuan, Sichuan, China	1640	102.90	30.80	This study
WL15250	IOZCAS	KU531752	KU531794	*KU531831*	KU531871	Wolong Nature reserve, Wenchuan, Sichuan, China	1750	102.90	30.80	This study
WL15306	IOZCAS	KU531753	KU531795	*KU531832*	KU531872	Wolong Nature reserve, Wenchuan, Sichuan, China	1450	102.90	30.80	This study
WL15334	IOZCAS	KU531754	KU531796	*KU531833*	KU531873	Wolong Nature reserve, Wenchuan, Sichuan, China	1500	102.90	30.80	This study
WL15356	IOZCAS	KU531755	KU531797	*KU531834*	KU531874	Wolong Nature reserve, Wenchuan, Sichuan, China	1600	102.90	30.80	This study
WL15361	IOZCAS	KU531756	KU531798	*KU531835*	KU531875	Wolong Nature reserve, Wenchuan, Sichuan, China	1525	102.90	30.80	This study
XZC002		GU479929*				Chawalong, Xizang, China		98.20	28.46	11
XZC004		GU479930*				Linzhi, Xizang, China		94.33	29.30	11
XZC006		GU479931*				Chawalong, Xizang, China		98.20	28.46	11
XZC007		GU479932*				Chawalong, Xizang, China		98.20	28.46	11
XZC010		GU479933*				Chawalong, Xizang, China		98.20	28.46	11
XZC011		GU479934*				Chawalong, Xizang, China		98.20	28.46	11
XZC012		GU479935*				Chawalong, Xizang, China		98.20	28.46	11
YJ002		HM037046				Yajiang, Sichuan,China		101.02	30.03	11
YNLC011	ICDC	KF740270	KF739983	KF739915	KF769371	Lincang Snow Mountain, Lincang, Yunnan, China	3200	100.03	23.90	This study
YNLC013	ICDC	KF740271	KU531777		KU531858	Lincang Snow Mountain, Lincang, Yunnan, China	3200	100.03	23.90	This study
YNLC015	ICDC	KF740272	KU531778		KU531859	Lincang Snow Mountain, Lincang, Yunnan, China	3200	100.03	23.90	This study
YNLC036	ICDC	KF740274	KU531779		KU531860	Lincang Snow Mountain, Lincang, Yunnan, China	2650	100.03	23.90	This study
YNLC063	ICDC	KF740275	KU531780		KU531861	Lincang Snow Mountain, Lincang, Yunnan, China	2650	100.03	23.90	This study
YNLC079	ICDC	KF740276	KU531781			Lincang Snow Mountain, Lincang, Yunnan, China	2500	100.03	23.90	This study

Number	Museum	CytB	COI	D-loop	IRBP	Location	Elevation	Longitude	Latitude	References
YNLJ032	IOZCAS	KU531742	KU531782	*KU531839*	KU531880	YuLong Mount, Lijiang, Yunnan, China	3264	100.00	27.00	This study
YN-ZD188	ICDC	KF740296	KF739986	KF739917	KF769390	Shangri-la, Yunnan,China	3650	99.00	27.00	This study
YN-ZD227	ICDC	KF740297	KU531802		KU531879	Shangri-la, Yunnan,China	3580	99.00	27.00	This study
YN-ZD266	ICDC	KF740298	KF739987	KF739918	KF769391	Shangri-la, Yunnan,China	3450	99.00	27.00	This study

Supplementary references

1. Zheng, S. H. *Quaternary rodents of Sichuan-Guizhou area, China.* (Science press, Beijing, 1993).
2. Zhang, Z., Liu, J., Zhang, H. & Yuan, C. A Pleistocene mammalian fauna from Panxian Dadong, Guizhou Province. *Acta Anthrop. Sinica* **16**, 209-220 (1997).
3. Li, C.L. & Xue, X.X. Discovery of rodents of Sichuan-Guizhou in Shaxi, Lantian. *Sci. Bull. China* **41**, 2071-2073 (1996).
4. Bacon, A.M. *et al*. Records of murine rodents (Mammalia, Rodentia) in the Pleistocene localities of Tan Vinh and Ma U'Oi (Northern Vietnam) and their implications to past distribution. *Ann. Paléont.* **92**, 367-383 (2006).
5. Wang, Y., Qin, D.G., Yang, Y. L., & Jin, C.Z. The early Pleistocene murid rodents from Juyuan Cave of Boyue Mountain, Chongzuo, Guangxi of south China. *Acta Anthrop. Sinica* **35**, 1-11 (2016).
6. Wu, X. Z., Deng, X. & Zheng, L. P. Late Pleistocene fauna at Yang'er cave in Hunan Province. *Quaternary Res.* **28**, 1114-1128 (2008).
7. Zou, S. L. *et al*. Preliminary report on the Late Pleistocene mammalian fauna from Shangli County, Pingxiang, Jiangxi Province. *Acta Anthrop. Sinica* **35**, 109-120 (2016).
8. Zhao, L.X. *et al*. New discovery of human fossils and associated mammal fauna from Mawokou Cave in Bijie, Guizhou Province of Southern China. *Acta Anthrop. Sinica* **35**, 24-35 (2016).
9. Jin, C.Z. *et al*. Preliminary report on the 2002 excavation of Jinpendong site at Wuhu, Anhui Province. *Acta Anthrop. Sinica* **23**, 281-291 (2004).

10. Jing, M., Yu, H.T., Wu, S.H., Wang, W. & Zheng, X. Phylogenetic relationships in genus *Niviventer* (Rodentia: Muridae) in China inferred from complete mitochondrial cytochrome b gene. *Mol. Phyl. Evol.* **44**, 521-529 (2007).
11. Chen, W. *et al*. Phylogeography of the large white-bellied rat *Niviventer excelsior* suggests the influence of Pleistocene glaciations in the Hengduan Mountains. *Zool. Scr.* **27**, 487-493 (2010).
12. Li, J. *et al*. DNA barcoding of Murinae (Rodentia: Muridae) and Arvicolinae (Rodentia: Cricetidae) distributed in China. *Mol. Ecol. Res* .**15**, 153-167 (2015).
13. Jansa, S. A., Barker, F.K. & Heaney,L. R. The pattern and timing of diversification of Philippine endemic rodents: evidence from mitochondrial and nuclear gene sequences. *Sys. Biol.* **55** (1), 73-88 (2006).
